# Investigation of pulmonary inflammatory responses following intratracheal instillation of and inhalation exposure to polypropylene microplastics

**DOI:** 10.1186/s12989-024-00592-8

**Published:** 2024-08-06

**Authors:** Taisuke Tomonaga, Hidenori Higashi, Hiroto Izumi, Chinatsu Nishida, Naoki Kawai, Kazuma Sato, Toshiki Morimoto, Yasuyuki Higashi, Kazuhiro Yatera, Yasuo Morimoto

**Affiliations:** 1 Department of Occupational Pneumology, Institute of Industrial Ecological Sciences, University of Occupational and Environmental Health, Japan, 1-1 Iseigaoka, Yahata-nishi-ku, Kitakyushu, Fukuoka 807-8555 Japan; 2 Department of Environmental Health Engineering, Institute of Industrial Ecological Sciences, University of Occupational and Environmental Health, Japan, 1-1 Iseigaoka, Yahata-nishi-ku, Kitakyushu, Fukuoka 807-8555 Japan; 3 Department of Respiratory Medicine, University of Occupational and Environmental Health, Japan, 1-1 Iseigaoka, Yahata-nishi-ku, Kitakyushu, Fukuoka 807-8555 Japan

**Keywords:** Microplastics, Polypropylene, Inhalation exposure, Intratracheal instillation, Pulmonary toxicity, Rat

## Abstract

**Background:**

Microplastics have been detected in the atmosphere as well as in the ocean, and there is concern about their biological effects in the lungs. We conducted a short-term inhalation exposure and intratracheal instillation using rats to evaluate lung disorders related to microplastics. We conducted an inhalation exposure of polypropylene fine powder at a low concentration of 2 mg/m^3^ and a high concentration of 10 mg/m^3^ on 8-week-old male Fischer 344 rats for 6 h a day, 5 days a week for 4 weeks. We also conducted an intratracheal instillation of polypropylene at a low dose of 0.2 mg/rat and a high dose of 1.0 mg/rat on 12-week-old male Fischer 344 rats. Rats were dissected from 3 days to 6 months after both exposures, and bronchoalveolar lavage fluid (BALF) and lung tissue were collected to analyze lung inflammation and lung injury.

**Results:**

Both exposures to polypropylene induced a persistent influx of inflammatory cells and expression of CINC-1, CINC-2, and MPO in BALF from 1 month after exposure. Genetic analysis showed a significant increase in inflammation-related factors for up to 6 months. The low concentration in the inhalation exposure of polypropylene also induced mild lung inflammation.

**Conclusion:**

These findings suggest that inhaled polypropylene, which is a microplastic, induces persistent lung inflammation and has the potential for lung disorder. Exposure to 2 mg/m^3^ induced inflammatory changes and was thought to be the Lowest Observed Adverse Effect Level (LOAEL) for acute effects of polypropylene. However, considering the concentration of microplastics in a real general environment, the risk of environmental hazards to humans may be low.

**Supplementary Information:**

The online version contains supplementary material available at 10.1186/s12989-024-00592-8.

## Background

Microplastics are defined as plastics with a diameter of 5 mm or less. They are produced by primary microplastics such as microscopic plastic beads and by secondary microplastics that have been miniaturized by crushing or degrading plastics. Marine plastic pollution, including microplastics, has become a problem in recent years, and the Organization for Economic Co-operation and Development (OECD) estimates that the amount of plastic leaked into the environment worldwide in 2019 was approximately 22 million tons. It is estimated that approximately 12% of the plastic is microplastics. The OECD estimates that by 2060, the amount of plastic waste will be approximately three-fold the current amount [1], and the amount of microplastics released into the environment will continue to increase, raising concerns about their influence on the environment and living organisms. Microplastics have also been observed in the atmosphere [2, 3]. They are released into the atmosphere from a variety of areas, including roads, sea spray, agricultural dust, dust from populated areas, and waste disposal facilities [4–6]. There is a report that polypropylene has the highest concentration of microplastics at the PM2.5 level in the atmosphere [7]. Polypropylene has been detected in the lungs of humans and birds, and it is indicated that polypropylene is taken in through breathing [8–10]. There is concern, therefore, about the biological effects of polypropylene due to inhalation exposure. It has also been reported that microplastics are generated in high concentrations not only in the general environment but also in occupational environments [11]. Polypropylene is widely used in food packaging and medical packaging, and is also a raw material in the manufacture of surgical masks. It is sufficiently conceivable that people may be exposed during the process of manufacturing plastic in an occupational environment. Therefore, it is also important to explore the Lowest Observed Adverse Effect Level (LOAEL) or No Observed Adverse Effect Level (NOAEL) of microplastics to estimate safe levels of exposure to microplastics.

Generally, the mechanism of lung disorder caused by inhalable chemicals is that they are deposited in the lungs and inflammation is triggered within the lungs. In other words, it is thought that persistent lung inflammation caused by macrophages and neutrophils causes irreversible fibrosis due to accelerated collagen deposition by fibroblasts, leading to the formation of lung tumors. These irreversible pathological conditions, such as fibrosis and tumorigenesis, are thought to be related to the retention of inhaled chemicals in the lungs [12, 13]. Considering that microplastics, which do not degrade easily, have high stability and a high retention capacity in the lungs, they may cause pathological conditions similar to lung disorders caused by inhalable chemicals.

In the present study, using polypropylene as a microplastic, we conducted an inhalation exposure, which is similar to the exposure route in humans, and an intratracheal instillation, which examines the potential of lung disorder by microplastics. We evaluated the biological effects of inhaling microplastics and the potential for lung disorders.

## Results

### Characterization of polypropylene aerosol and suspension

**Fig. 1:**
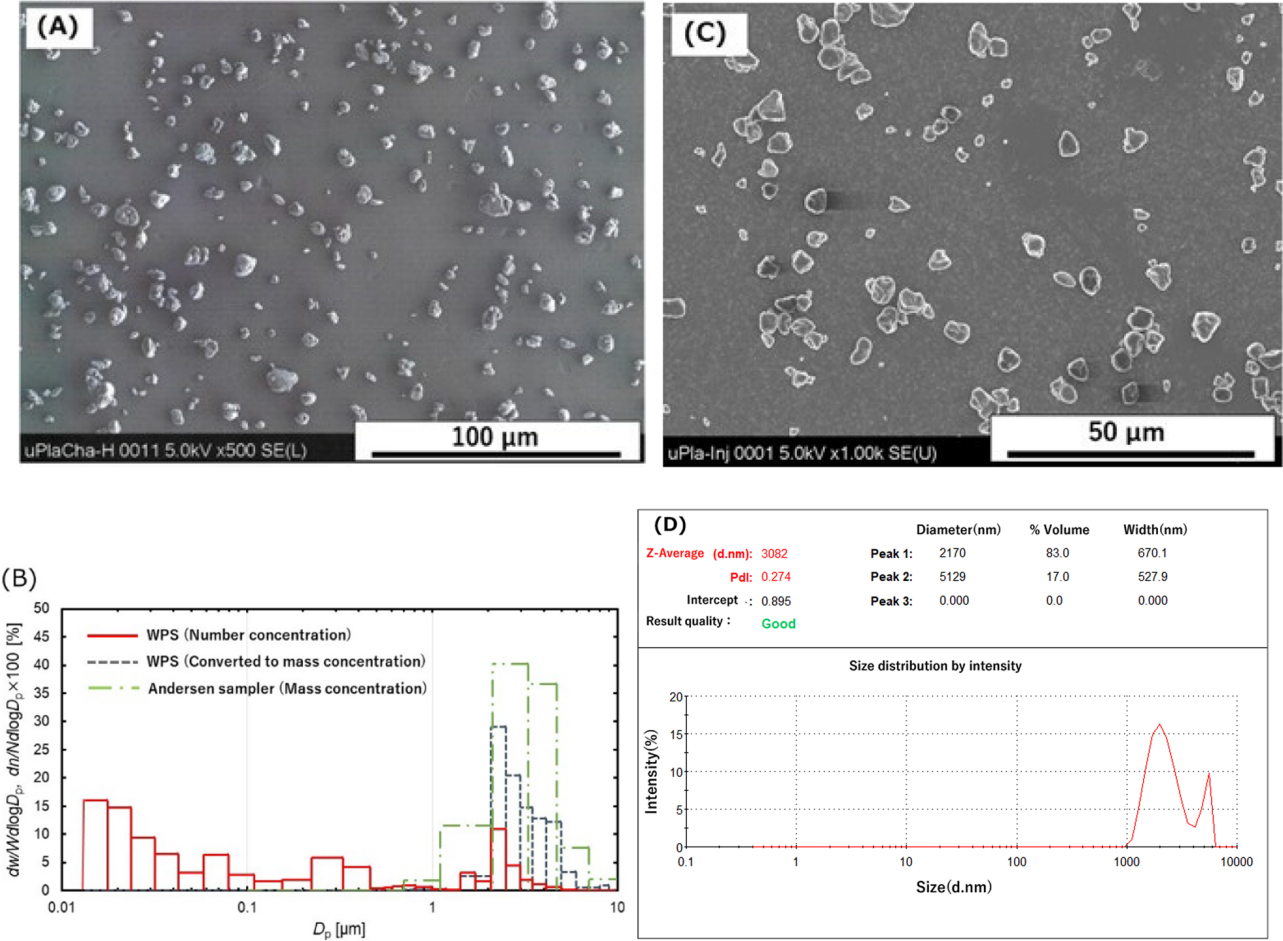
Characterization of polypropylene aerosol and suspension. SEM observation of polypropylene aerosol (**A**). Particle size distribution of polypropylene in aerosol (**B**). SEM observation of polypropylene suspension (**C**). Particle size distribution of polypropylene in suspension by dynamic light scattering (**D**)

The SEM image of high concentrations of polypropylene aerosols in the inhalation chamber was dispersed and their size was between 1 µm and 10 µm (Fig. 1A). Bimodal peaks on the nanoscale and micron-scale were detected in the inhalation chamber in the number concentration distribution by WPS. The trends of the WPS translated weight concentration conversion and the weight concentration distribution measured by Andersen Sampler were consistent (Fig. 1B). The Mass median aerodynamic diameter of the polypropylene in the exposure chamber measured by Andersen Sampler was 3.0 µm (Geometric Standard Deviation (GSD) = 1.7). It was confirmed that a stable concentration could be generated in the chambers during the exposure period (Additional file: Figure S1).

The SEM images of the polypropylene suspension show well dispersion in the high dose (Fig. 1C). There was also a bimodal peak in the particle size distribution of polypropylene in the suspension, and the agglomerate average diameter was 3.082 µm as measured by DLS (Fig. 1D).

### Inflammatory response following inhalation exposure to polypropylene

**Fig. 2:**
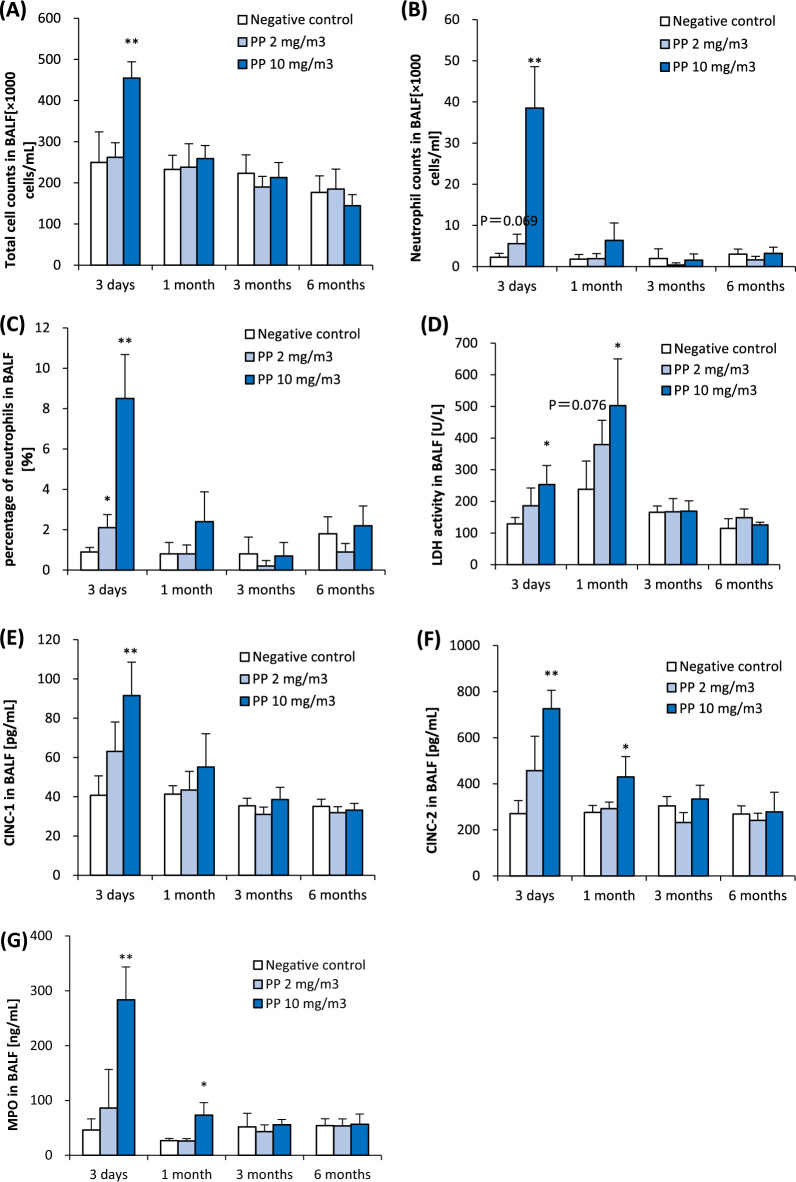
Cell analysis and cytokine concentration in BALF following inhalation exposure of polypropylene. Total cell counts in BALF (**A**). Neutrophil count in BALF (**B**). Percentage of neutrophils in BALF (**C**). LDH activity in BALF (**D**). Concentration of CINC-1 in BALF (**E**). Concentration of CINC-2 in BALF (**F**). Concentration of HO-1 in BALF (**G**). Inhalation exposure of polypropylene induced persistent influx of inflammatory cells and expression of CINC-1, CINC-2, and MPO in BALF from 1 month after exposure. Data are presented as mean ± SD for n = 5/group (*p < 0.05, **p < 0.01)

Regarding cell analyses in bronchoalveolar lavage fluid (BALF), the total cell counts in BALF were significantly increased only after 3 days in the high-concentration group compared to the negative control group (Fig. 2A). The neutrophil counts in BALF showed a significant increase from 3 days to 1 month in the high-concentration group, and a nearly significant increase was observed at only 3 days after exposure in the low-concentration group (p.0.069) (Fig. 2B). A significant increase in the percentage of neutrophils in BALF was observed in both the low-concentration group and the high-concentration group 3 days after exposure, and a significant persistent increase was observed in the high-concentration group at 1 month after exposure (Fig. 2C). Comparing the results of our previous 4-week inhalation exposure tests of nanomaterials [14, 15], polypropylene caused less inflammation than nickel oxide nanoparticles, which are known to be highly toxic to the lungs and, had the same level of inflammation as titanium dioxide nanoparticles, which have low toxicity to the lungs (Additional file: Figure S2A). Regarding inflammatory markers in BALF, there was a persistent increase of LDH activity in BALF for up to 1 month in the high-concentration group (Fig. 2D). CINC-1 and CINC-2 are involved in neutrophil migration. The CINC-1 concentration in BALF significantly increased in the high-concentration group at 3 days after exposure, and the CINC-2 and MPO concentrations in BALF were significantly increased from 3 days to 1 month in the high-concentration group (Fig. 2E–G). No significant differences in rat body weight were observed following inhalation exposure (Additional file: Figure S3A).

### Inflammatory response following intratracheal instillation of polypropylene

**Fig. 3:**
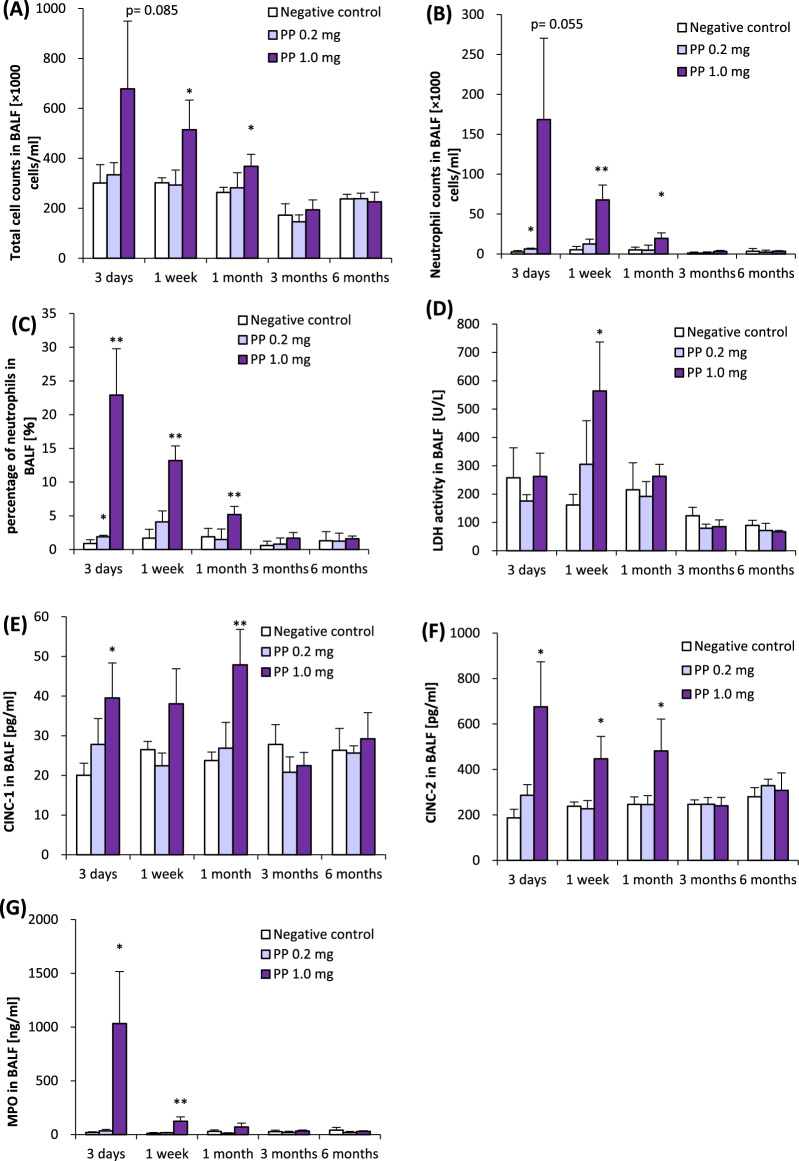
Cell analysis and cytokine concentration in BALF following intratracheal instillation of polypropylene. Total cell counts in BALF (**A**). Neutrophil count in BALF (**B**). Percentage of neutrophils in BALF (**C**). LDH activity in BALF (**D**). Concentration of CINC-1 in BALF (**E**). Concentration of CINC-2 in BALF (**F**). Concentration of HO-1 in BALF (**G**). Inhalation exposure of polypropylene induced persistent influx of inflammatory cells and expression of CINC-1, CINC-2, and MPO in BALF from 1 month after instillation. Data are presented as mean ± SD for n = 4–5/group (*p < 0.05, **p < 0.01)

Regarding cell analyses in BALF, the total cell count, neutrophil count, and percentage of neutrophils in BALF were persistently increased for up to 1 month in the high-concentration group. Even in the low-dose group, the neutrophil count and percentage of neutrophils showed a significant increase only after 3 days, and there was lung inflammation by polypropylene in a concentration-dependent manner (Fig. 3A–C). Comparing the results of our previous intratracheal instillation of nanomaterials [14, 15], polypropylene caused less persistent inflammation than nickel oxide nanoparticles, which are known to be highly toxic to the lungs, but persistent lung inflammation by polypropylene was observed compared to titanium dioxide nanoparticles and zinc oxide nanoparticles, which cause only transient lung inflammation (Additional file: Figure S2B). LDH activity in BALF was significantly increased in the high-dose group compared to the negative control group at only 1 week after instillation (Fig. 3D). CINC-1 and CINC-2 concentrations significantly increased from 3 days to 1 month in both high-dose groups (Fig. 3E, F). The MPO concentration in BALF significantly increased in the high-dose group from 3 days to 1 week, and a tendency of increase of MPO was observed in the high-dose group at 1 month after installation (Fig. 3G). No significant differences in rat body weight were observed following intratracheal instillation (Additional file: Figure S3B).

### Histopathological findings

**Fig. 4:**
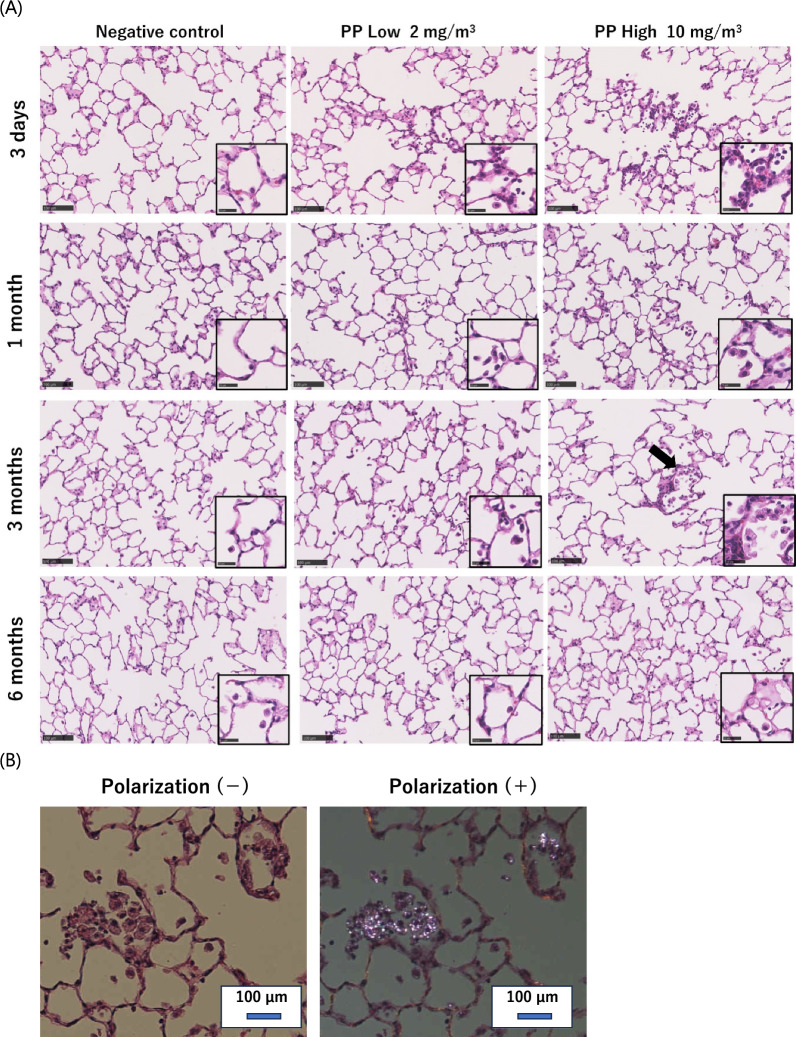
Hematoxylin and eosin staining of lung sections following inhalation exposure of polypropylene at each time course (**A**), and comparison of polarized light observation in the lung at 3 months after exposure in the high concentration group (**B**). Persistent mild inflammation, mainly neutrophils and alveolar macrophages, was observed until 1 month after exposure. Aggregation of inflammatory cells was observed at 3 months after exposure in the high-concentration group (Black arrow). (Scale bar: 100 µm)

Inhalation study (Fig. 4): In the inhalation exposure in the high concentration group, an influx of inflammatory cells into the alveolar space was observed until 3 months after exposure; in particular, an aggregation of inflammatory cells was observed at 3 months after exposure. Polarized light microscopy revealed that the aggregated inflammatory cells contained polarized material, suggesting that the inflammatory cells, mainly macrophages, phagocytosed polypropylene and aggregated it. In the low-concentration group, a very slight influx of inflammatory cells into the alveolar spaces was observed at 3 days after exposure. No fibrosis was detected in either group throughout the observation time.

**Fig. 5:**
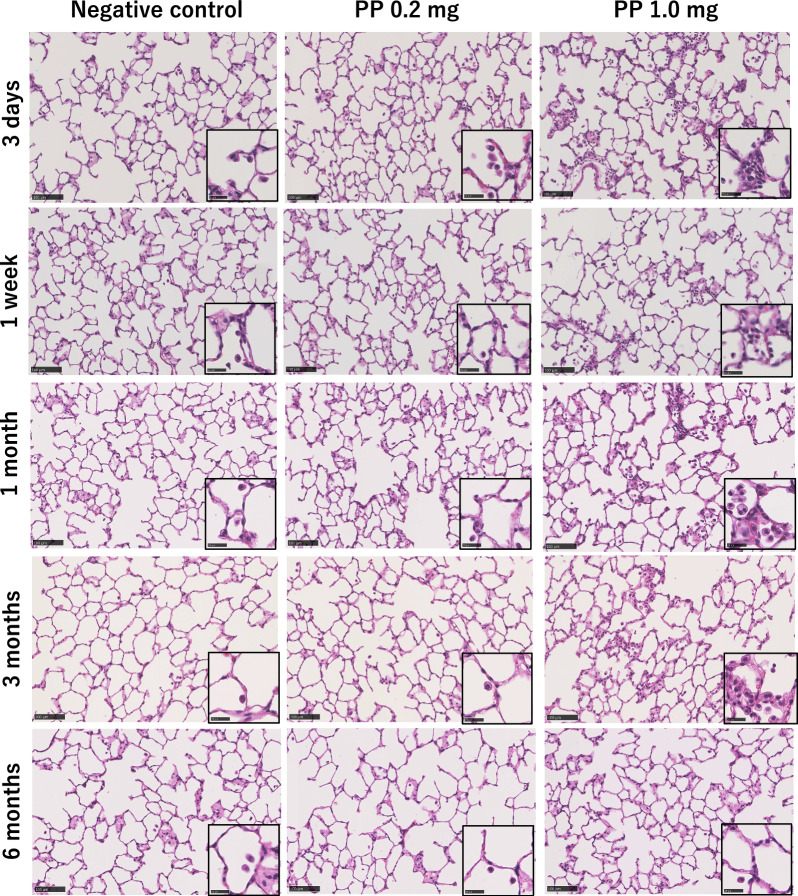
Hematoxylin and eosin staining of lung sections following intratracheal instillation of polypropylene at each time course. Persistent mild inflammation, mainly neutrophils and alveolar macrophages, was observed until 3 months after exposure. (Scale bar: 100 µm)

Intratracheal instillation study (Fig. 5): In the high-dose group, an influx of inflammatory cells into the alveolar space was observed from 3 days to 1 month after instillation, and in the low-dose group, there was a very slight influx of inflammatory cells into the alveolar space at only 3 days after instillation. No fibrosis was observed in either group.

### Microarray analysis and KEGG pathway analysis

**Fig. 6:**
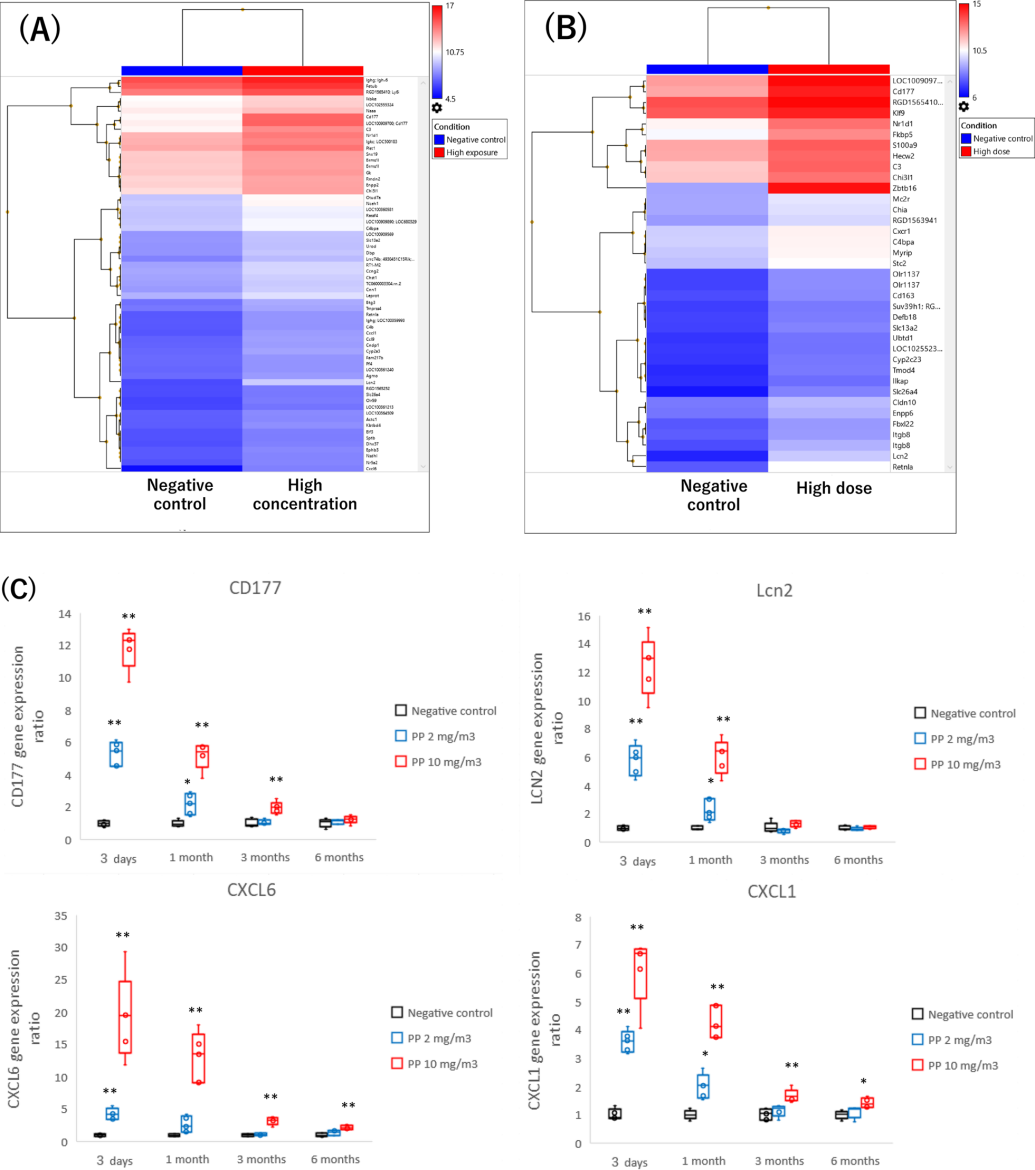
Results of microarray and Quantitative real-time polymerase chain reaction Quantitative real-time polymerase chain reaction (qRT-PCR) on genes that were highly expressed in the microarray analysis in lung tissue after inhalation exposure of polypropylene. Clustered heatmaps showing the expression patterns based on mRNA whose expression ratio is more than twice that of the control among genes whose expression level is above the median value in inhalation exposure (**A**) and intratracheal instillation (**B**), respectively. qRT-PCR in lung tissue in inhalation exposure (**C**). Red and blue represent high and low expression in each exposure group, and color density indicating levels of fold change was displayed in the heatmaps. Persistent increases of genes at 1–6 months after exposure in the high-concentration group in qRT-PCR analysis. Data are presented as mean ± SD for n = 5/group (*p < 0.05, **p < 0.01)

We conducted a microarray analysis of mRNA in the lung tissue at 1 month after the inhalation exposure and intratracheal instillation. In the inhalation exposure, among the 11,594 genes that were found to be expressed more than the median, 64 genes were detected to be more than twice as expressed compared to the negative control group, and increased expression of genes related to chemokines and lung inflammation was observed (Fig. 6A). In the intratracheal instillation, among the 11,594 genes that were found to be expressed, 37 genes were detected to be more than twice as expressed compared to the control group, and an increased expression of genes related to chemokines and lung inflammation was observed (Fig. 6B). An increased expression of the top 10 genes expressed in the inhalation exposure was also observed in a microarray analysis of the intratracheal instillation, and there were similar trends in gene expression in the inhalation exposure and the intratracheal instillation (Table 1).

**Table 1:** Comparison of TOP of 10 gene expression after inhalation exposure and gene expression after intratracheal instillation

Gene symbol	Gene description	Inhalation exposure (Fold charge)	Intratracheal instillation (Fold charge)
Cd177	CD177 molecule	12.45	5.31
LOC100909700*	CD177 antigen-like	10.91	6.19
Lcn2	lipocalin 2	9.04	7.71
Cxcl6	Chemokine (C-X-C motif) ligand 6	7.05	2.98
C3	Complement component 3	6.06	3.55
Cxcl1	Chemokine (C-X-C motif) ligand 1	3.36	1.94
Otud7a	OTU deubiquitinase 7A	3.05	1.41
Ccl9	Chemokine (C–C motif) ligand 9	2.86	1.51
Nceh1	Neutral cholesterol ester hydrolase 1	2.84	1.02
Retnla	Resistin like alpha	2.77	7.63

*LOC100909700 (tentatively renamed CD177 in the rat genome database)

To evaluate the effects on the lungs of inhaling polypropylene, we performed a pathway analysis using the 64 genes whose expression was more than twice that of the negative control group in inhalation exposure. We extracted pathways related to cytokines and inflammation, such as the IL-17 signaling pathway, chemokine signaling pathway, and cytokine-cytokine receptor interaction (Table 2).

**Table 2:** Result of KEGG pathway analysis in inhalation exposure

Terms of KEGG pathway	Gene Counts	%	*P* Value	Benjamini
Pertussis	4	8.33	0.0014	0.070
Viral protein interaction with cytokine and cytokine receptor	4	8.33	0.0021	0.070
IL-17 signaling pathway	4	8.33	0.0027	0.070
Alcoholic liver disease	4	8.33	0.0078	0.154
Chemokine signaling pathway	4	8.33	0.0175	0.276
Kaposi sarcoma-associated herpesvirus infection	4	8.33	0.0273	0.323
Complement and coagulation cascades	3	6.25	0.0286	0.323
Cytokine-cytokine receptor interaction	4	8.33	0.0485	0.479

### Analysis of quantitative real-time polymerase chain reaction

We performed quantitative real-time polymerase chain reaction (qRT-PCR) on genes that were highly expressed in the microarray analysis of the inhalation exposure (Fig. 6C). CD177, which is expressed on the surface of neutrophils and is involved in neutrophil migration, remained significantly elevated in the high-concentration group compared to the negative control group up to 3 months after exposure, and even in the low-concentration group a persistent increase was observed until 1 month later. LCN2, which regulates innate immunity such as neutrophil recruitment, was significantly increased in both the low and high-concentration groups until 1 month after exposure. A significant increase in the chemokines CXCL1 and CXCL6 was observed in the high-concentration group up to 6 months after exposure, and a significant increase was also observed in the low-concentration group from 3 days to 1 month. An analysis of the expression levels of major inflammatory cytokines revealed no significant increases in IL-1β, IL-4, or IL-13 compared to the negative control group. A transient increase in IL-5 was observed in the high-concentration group after 3 months (Figure S2). There was no significant increase in IL-17a gene expression compared to the control group, but an increasing trend was observed at 3 days and 1 month after exposure.

## Discussion

The biological effects of inhalable chemicals can be broadly divided into two types by time course: acute effects and chronic effects. To understand what pathological conditions are caused by the acute effects is important basic data for considering the setting of exposure limits and ceiling values, and for evaluating the chronic effects. In addition, NOAEL and LOAEL obtained through animal exposure examination are used as the basis for setting Threshold Limit Values and Environmental Quality Standards [16–18]. To estimate LOAEL and NOAEL in animal exposure examination, it is necessary to conduct appropriate exposure, which has exposure concentrations at which biological effects can be observed. Therefore, the concentrations in animal exposure examination may be higher than in the real environment. In the inhalation exposure of this study, polypropylene fine particles were used, a low concentration of 2 mg/m^3^ and a high concentration of 10 mg/m^3^ were set, and a short-term inhalation exposure for 4 weeks was conducted to investigate the acute effects. These exposure concentrations were referred to the reports of respiratory symptoms in workers handling polypropylene (exposure concentration 4.4 mg/m^3^) [11] and referring to the previous inhalation exposure we have conducted on inorganic substances [14, 15, 19] to be those at which biological effects are recognized, which are necessary for determining NOAEL and LOAEL. The analysis of inflammatory cells in BALF and observation of pathological lung findings revealed that, in the inhalation exposure, there was persistent lung inflammation for 1 month after exposure in the high-concentration group, and also transient and very mild inflammatory changes at 3 days after exposure in the low-concentration group, indicating acute phase inflammatory changes.

It has been reported that inhalation exposure studies have been conducted on laboratory animals with other air pollutants, and acute lung inflammation has been caused [20–22]. Acute phase lung inflammation was observed in an inhalation exposure (exposure concentration: 1.69 ± 0.67 mg/m^3^) of carbon black, which simulates microparticles in urban air [20]. Regarding microplastic inhalation exposure, there have been no reports on polypropylene, but increases in inflammatory cytokines and neutrophils have been reported for polystyrene and nylon (polyamide) [21, 22]. Inhalation exposure to polypropylene, a microplastic, is thought to cause lung inflammation upon acute exposure, similar to these air pollutants and other microplastics.

The exposure dose for the intratracheal instillation in this study was set at a low dose of 0.2 mg/rat and a high dose of 1.0 mg/rat. Although the intratracheal instillation doses in this study are likely higher than the actual environmental amounts, the potential for lung disorders was evaluated by intratracheal instillation to compare with other inhalable chemicals. There is the root fact from previous intratracheal instillation studies that 0.2 mg is the minimum dose that causes inflammation with highly toxic particles [23] and that even less toxic particles cause persistent lung inflammation when the dose exceed 1.0 mg [24, 25]. Under these exposure conditions, we previously found that inhalable chemicals with high lung toxicity caused inflammation and fibrosis that persisted for more than 3 months, while ones with low lung toxicity were recovered from within 1 month. Therefore, the borderline for evaluating lung disorders is considered to be the presence or absence of persistent inflammation for 1 to 3 months [14, 19, 26, 27].

In the intratracheal instillation of polypropylene, the number of neutrophils in BALF showed a persistent increase up to 1 month after instillation in the high-dose group and a transient increase only 3 days after instillation in the low-dose group. Observation of histopathological findings in lung showed an influx of inflammatory cells into the alveolar spaces up to one month after instillation, but no inflammatory cell infiltration into the alveolar interstitium was observed. It has been reported that even when inhalable chemicals with high toxicity such as asbestos and crystalline silica are instilled into the trachea, they have inflammatory potential that lasts for more than a month [28–30]. In multiple intratracheal instillation studies of microplastics, intratracheal instillation of polystyrene or polypropylene has been shown to increase neutrophils and inflammatory cytokines in the acute phase [31, 32]. If the mechanism of lung disorders caused by microplastics is similar to that caused by inorganic chemicals, then the polypropylene in this study, having caused persistent lung inflammation for up to 1 month, indicates the persistence of lung inflammation, and we considered that the potential of lung disorder was not low.

The results of the inhalation exposure in this study indicated that polypropylene is a material that causes acute lung inflammation. In the intratracheal instillation, it was indicated that the potential for lung disorders by polypropylene may not be low. The amount of atmosphere microplastics in the real environment is reported to be 238 ng/m^3^ at PM2.5 size [33]. The number concentration is affected by the measurement location, conditions, and analysis method, but it has been reported to range from a few to several thousand particles/m^3^ [2, 3, 34]. Exposure concentrations under experimental conditions are high compared to the abundance of microplastics in the atmosphere; therefore, it is difficult at present to imagine that exposure at the concentration in this study would occur in the atmosphere. Considering the concentration of microplastics in a real general environment, the risk of environmental hazards to humans may be low. However, human exposure at the mg/m^3^ level has been reported in occupational environments [11, 35], It is thought that there is potential for human exposure in the process of manufacturing plastic, recycling plastic, or fusing plastics [36]. Also, in the case of an accidental event occurring at an industrial waste processing facility, such as a plastic processing facility, it can be assumed that a large amount of microplastics will be generated, and it is necessary to consider an assessment of the acute effect of microplastics.

It is also important to note that there are various types of microplastics in the atmosphere, and it is difficult to judge various microplastics in the atmosphere as a single type of microplastic because there are no standardized microplastics in the atmosphere [2, 3]. Microplastics in the real environment exist in various states due to degradation by sunlight, weathering, coexistence with other chemicals, and adhesion [2, 37]. It is ideal to collect and expose microplastics in the real environment. However, exposure testing is difficult to conduct in laboratory exposures because of the large amount of microplastics required. Therefore, inhalation exposure and intratracheal instillation in this study were performed using a single crushed, untreated microplastic. In the future, it will be necessary to analyze microplastics with various treatments, such as photodegradation, to evaluate the biological effects of microplastics closer to the real general environment.

In this study, we examined the expression of chemokines that promote neutrophil migration and activation as a mechanism of pulmonary inflammation. CINC-1 (CXCL1) and CINC-2 (CXCL3), which are mainly produced by macrophages, are known to activate neutrophils and function as chemoattractants [38, 39]. In the inhalation exposure, there were significant persistent increases of CINC-1 and CINC-2 in the high-concentration group until one month after exposure. In the intratracheal instillation, there were significant increases of CINC-1 and CINC-2 until 1 month after instillation, similar to the tendency of neutrophil counts in BALF in inhalation and intratracheal instillation. It has been reported that the increase in CINCs and neutrophil inflammation are linked to the intratracheal instillation of inorganic nanomaterials and bleomycin [14, 40, 41]. Woo et al. have reported an increase in CXCL1 and neutrophil counts in an intratracheal instillation of polypropylene nanoparticles [42]. It is thought that, with exposure to microplastics in the lungs, CINCs are released mainly from macrophages, and neutrophils are migrated and activated.

MPO is an enzyme produced by neutrophils that catalyzes hydrogen peroxide to generate hypochlorous acid (HOCl), a powerful free radical, which is involved in lung injury and lung inflammation [43]. In our inhalation exposure, a tendency of persistent increase of MPO was observed until one month after exposure. Even with intratracheal instillation, a persistent increase was observed up to 1 month after instillation. A correlation among MPO, neutrophils, and CINC have been observed in inhalation exposure and intratracheal instillation of nanomaterials, which are inorganic substances [26]. Considering that microplastics also have the same tendency to increase neutrophils, CINC, and MPO, there is thought to be a series of processes whereby CINC release promotes neutrophil migration and the activated neutrophils cause lung disorders in the effect of microplastics on the lungs, similar to inorganic chemicals.

In this study, we conducted a comprehensive genetic analysis of the lungs, which has rarely been performed in the analysis of microplastic-induced lung inflammation, and explored the expression of candidate genes related to polypropylene-induced lung inflammation. Among the 11,594 genes that were found to be expressed in the lung tissue samples one month after the inhalation exposure, there were 64 genes whose expression levels were above the median in the high-concentration exposure group and twice as high as those in the control group, and these genes were mainly related to chemokines and lung inflammation. Expression of the genes listed in the inhalation exposure was also observed in the intratracheal instillation (Table 2). In the inhalation exposure, the gene expression of CD177 were the most elevated. CD177 is a glycosyl phosphatidylinositol (GPI)-linked N-glycosylated transmembrane protein expressed primarily on the surface of neutrophils and has been reported to regulate neutrophil migration and activation [44–46]. In severe coronavirus disease 2019 (COVID-19), which is characterized by an increase in neutrophils and inflammatory cytokines, it has been reported that CD177 levels in serum correlate with the severity of the disease [46].

Lipokine 2 (LCN2), also known as neutrophil gelatinase-associated lipocalin (NGAL), which has been suggested as a biomarker of kidney injury [47], regulates innate immunity such as neutrophil recruitment [48], and it has also been reported to be associated with various lung diseases and infections [49]. Increased levels of LCN2 have also been reported in bleomycin-induced lung inflammation and tobacco exposure that causes neutrophilic inflammation [49, 50]. In addition, CXCL6 is a chemokine with neutrophil chemotaxis, and it has been reported that CXCL6 expression is observed in acute lung inflammation caused by bleomycin administration and lung injury caused by nanomaterials [51, 52]. Nishida et al. have reported that a persistent increase in the expression of CXCL6 in nanomaterials is useful as a marker for predicting lung disorders [52]. Considering that CXCL6 expression kept increasing for up to 6 months in the high concentration group in this study, we can conclude that polypropylene may cause lung disorder. The results of these gene expression analyses indicate that the lung inflammation caused by polypropylene is mainly caused by neutrophilic inflammation and that pulmonary inflammation is caused by the migration and activation of neutrophils following the release of the chemokine as CINCs.

Pathway analysis of genes whose expression was observed to be increased in the inhalation exposure revealed Chemokine and IL-17 pathways (Table 2). IL-17 is known to promote the chemotaxis of neutrophils by promoting the release of IL-8 (CINC-1 in rats) from macrophages and lung fibroblasts [53]. In the qRT-PCR analysis in this study, the gene expression of IL-17a was not significantly increased in the high-concentration exposure group, but an upward trend was observed, and there was a significant increase in the expression of the chemokines CXCL1 and CXCL6 (Fig. 6, Figure S4). Woo et al. have reported that polypropylene intratracheally instilled into mice at up to 5 mg/kg, 5 days a week for 4 weeks induced oxidative damage in intracellular mitochondria to induce IL-8 via p-38-NF-kB. They also stated that the expression of inflammatory cytokines related to the IL-17 pathway was increased [42]. Considering that the p38-mediated transcription factor NF-kB leads to the induction of chemokines in the IL-17 pathway [54], these results of comprehensive genetic analysis and qRT-PCR in this study suggest that the IL-17 pathway may be involved in neutrophilic lung inflammation in the inhalation exposure of polypropylene.

## Conclusion

In this study, we conducted an inhalation exposure and an intratracheal instillation in rats using polypropylene as a microplastic. In both exposures, neutrophilic lung inflammation was the main pathological condition. In the inhalation exposure, microplastics showed lung inflammation in a dose-dependent manner. In the low concentrations of exposure, there was a transient increase of neutrophil at 3 days after exposure and genetic changes up to 1 month after exposure, and even at high concentrations, there were persistent neutrophil-induced changes. Based on these results, the low concentration in the inhalation exposure is considered to be LOAEL for acute effects, but since no lung injury was observed, the exposure of the low concentration is considered to be close to the NOAEL. Considering the concentration of microplastics in a real general environment, the risk of environmental hazards of microplastics to humans may be low. In the intratracheal instillation, on the other hand, persistent lung inflammation was observed, and it is thought that the lung disorder was not low. The results of this study therefore suggest that it is necessary to examine the chronic effects of microplastics in inhalation exposure.

## Methods

### Sample preparation

A sample of Polypropylene was purchased from SEISHIN ENTERPRISE Co.,Ltd. (PPW-5 J, Tokyo, Japan). Polypropylene is a white, easily scattered powder, with a density of 0.89 (20 ℃). No additives are used in the process of synthesizing polypropylene. Images of scanning electron microscopy (SEM) by HITACHI S-4500 (Hi-tachi, Ltd., Tokyo, Japan) of polypropylene were obtained by sampling aerosol in an inhalation chamber and by drying the dispersion, respectively. Polypropylene suspended in 0.4 ml distilled water including 0.1% Tween 80 was used for the intratracheal instillation. The size distribution of the polypropylene particles in the dispersion was measured by Dynamic Light Scattering (DLS) (ZEN1600, Malvern Panalytical, Ltd., Malvern, UK) and a dried sample of the dispersion was observed by SEM.

### Animals

Male Fischer 344 rats (Inhalation exposure: 6 weeks old, Intratracheal instillation: 10 weeks old) were purchased from Japan SLC, Inc. (Shizuoka, Japan). The animals were kept in the Laboratory Animal Research Center of the University of Occupational and Environmental Health for 2 weeks with access to free-feeding of commercial diet and water. All procedures and animal handling were done according to the guidelines described in the Japanese Guide for the Care and Use of Laboratory Animals as approved by the Animal Care and Use Committee, University of Occupational and Environmental Health, Japan (animal studies ethics clearance proposal number; AE20-015 and AE22-023).

### Inhalation exposure

The inhalation system consisted of a dust generator, exposure chambers (volume; 0.57 m^3^), and gas–liquid-solid separators, as described by Tanaka et al. [55]. To obtain a constant concentration, the polypropylene was mixed with fluidizing particles (small glass beads with a diameter of 250 μm) and polypropylenes close to each other were prevented from agglutinating. The mixture was fed into a hopper and transported smoothly via a continuous screw feed into a fluidized bed. Dry air flow was blown through the fluidized bed to transport the polypropylene to the exposure chamber while leaving behind the fluidizing particles. Only the glass beads remained in the fluidized bed because they were heavier than the polypropylene. The polypropylene aerosol weight concentration in the exposure chamber was measured daily by the isokinetic suction of air though a glass fiber filter beside the chamber. The glass fiber filter was weighed before and after the measurements in accordance with the standard method in JIS-Z8808, and the measured daily exposure weight concentration was determined as mg/m^3^. The particle size distribution for number concentration of the aerosols inside the exposure chamber was also measured in the diluted condition repeatedly using a particle size spectrometer (model 1000XP WPS, MSP Corp., Shoreview, MN). The weight concentration distribution of polypropylene in the inhalation chamber was measured by an Andersen Sampler (AN-200, Dylec Inc., Ibaraki, Japan). The sampling using a membrane filter was also performed to off-line analysis using SEM. After exposure for 4 weeks, the rats were dissected at 3 days, 1 month and 3 months, under anesthesia by isoflurane (VIATRIS Japan, Tokyo, Japan) inhalation.

### Intratracheal instillation

Doses of 0.2 mg (0.8 mg/kg BW) and 1.0 mg (4.0 mg/kg BW) of polypropylene suspended in 0.4 ml distilled water including 0.1% Tween 80 were administered to the lungs of rats (12 weeks old) in single intratracheal instillations. The rats were intratracheally instilled under anesthesia by sevoflurane (VIATRIS Japan, Tokyo, Japan) inhalation. Briefly, a laryngeal extension was performed using a laryngoscope blade (MAC1, Rudolf Riester GmbH, Jungingen, Germany), and an animal feeding needle (KN-348, Natsume Seisa-kusho Co., Ltd., Tokyo, Japan) was inserted directly into the trachea, and the suspension was manually injected. Then, 3 ml of air twice with a syringe from the animal feeding needle was inserted into the trachea. The rats were then allowed to awaken spontaneously and were observed periodically. The control group received distilled water including 0.1% Tween 80 water suspension. The rats were dissected at 3 days, 1 week, 1 month, 3 months and 6 months after the instillation under anesthesia by isoflurane (VIATRIS Japan, Tokyo, Japan) inhalation.

### Animals following inhalation and intratracheal instillation

There were 5 rats in each exposure and control group at each time point. Body and lung weights were measured under anesthesia by isoflurane (VIATRIS Japan, Tokyo, Japan) inhalation, and then, at autopsy, blood was removed from the abdominal aorta and the lungs were perfused with normal saline. The right lungs were repeatedly inflated with normal saline under a pressure of 20 cm H_2_O, following fluid recovery two times, while the left main bronchus was clamped. Between 7 and 14 mL of the recovered fluid (BALF) was collected in collection tubes by free fall, and then the right and left lungs were divided. The homogenized third lobes of the right lungs were used for cDNA microarray after recovery of BALF. The left lungs were inflated and fixed by 10% formaldehyde under a pressure of 25 cm H_2_O for use in histopathological evaluation.

### Cytospin analysis of inflammatory cells and measurement of inflammation related markers in BALF

BALF was centrifuged at 400 g at 4 °C for 15 min, and the supernatant was transferred to a new tube for measurement of total protein, lactate dehydrogenase (LDH) and cytokines. The pellets were washed by suspension with polymorphonuclear leukocyte (PMN) Buffer (137.9 mM NaCl, 2.7 mM KCl, 8.2 mM Na_2_HPO_4_, 1.5 mM KH_2_PO_4_ and 5.6 mM C_6_H_12_O_6_) and centrifuged at 400 g at 4 °C for 15 min. After removal of the supernatant, the pellets were resuspended with 1 mL of PMN (poly-morphonuclear leukocyte) Buffer. The number of cells in the BALF was counted by ADAM-MC (AR BROWN CO., LTD, Tokyo, Japan), and the cells were splashed on a slide glass using cytospin, and fixed and stained with Diff-Quik (Sysmex CO., Kobe, Hyogo, Japan), after which the number of neutrophils and alveolar macrophages were counted by microscopic observation. The neutrophil count was calculated by multiplying the total cell counts by the neutrophil ratio from the counting of them. The released LDH activity in the BALF supernatant was measured by a Cytotoxicity Detection KitPLUS (LDH) (Roche Diagnostics GmbH, Mannheim, Nordrhein-Westfalen, Germany) according to the manufacturer’s instructions. LDH activity was estimated using a standard curve obtained from known concentrations of recombinant LDH from rabbit muscle (Oriental Yeast Co., ltd., Tokyo, Japan). Concentrations of CINC-1 and CINC-2 in the BALF were measured by ELISA kits, #RCN100, #RCN200 (R&D Systems, Minneapolis, MN, USA), ELISA kits, respectively. The concentrations of rat MPO proteins in the BALF samples in all of the examinations were measured by ELISA kits, HK105 (Hycult Biotech, The Netherlands). All measurements were performed according to the manufacturer’s instructions.

### Total RNA extraction

The third lobes of the right lungs (n = 5 per group per time point) were homogenized while using a QIAzol lysis reagent with a TissueRupotor (Qiagen, Hilden, Ger-many). Total RNA from the homogenates was extracted using a miRNeasy Mini Kit (Qiagen, Hilden, Germany) following the manufacturer’s instructions. RNA purity and integrity were evaluated by ND-1000 Spectrophotometer (NanoDrop, Wilmington, USA), Agilent 2100 Bioanalyzer (Agilent Technologies, Palo Alto, USA).

### Affymetrix whole transcript expression arrays methods

The Affymetrix Whole Transcript Expression array process was executed according to the manufacturer’s protocol (GeneChip Whole TranscriptPLUS reagent Kit). cDNA was synthesized using the GeneChip WT (Whole Transcript) Amplificationkit as described by the manufacturer. The sense cDNA was then fragmented and biotin-labeled with TdT (terminal deoxynucleotidyl transferase) using the GeneChip WT Terminal labeling kit. Approximately 5.5 μg of labeled DNA target was hybridized to the Affymetrix GeneChip Rat Clariom-Schip at 45 °C for 16 h. Hybridized arrays were washed and stained on a GeneChip Fluidics Station 450 and scanned on a GCS3000 Scanner (Affymetrix). Signal values were computed using the Affymetrix® GeneChip™ Command Console software. We created a heatmap using Transcriptome Analysis Console (TAC) version 4.0 (Thermo Fisher Scientific Inc., Waltham, MA, USA). We analyzed the related genes that were found to be elevated in the polypropylene using the above microarray data for Kyoto Encyclopedia of Genes and Genomes (KEGG) Pathway analysis via the Database for Annotation, Visualization and Integrated Discovery (DAVID) version 2021 (https://david.ncifcrf.gov/home.jsp).

### Validation of gene expression data using quantitative real-time polymerase chain reaction

Quantitative real-time polymerase chain reaction (qRT-PCR) was performed as described previously [15]. Briefly, the total RNA extracted from the lungs at each observation point in each group was transcribed into cDNA (High-Capacity cDNA Reverse Transcription Kit, Thermo Fisher Scientific Inc., MA, USA). qRT-PCR assays were performed while using TaqMan (TaqMan Gene Ex-pression Assays, Thermo Fisher Scientific Inc., Waltham, MA, USA) according to the manufacturer’s protocol. Gene expression data were analyzed by the comparative cycle time (ΔΔCT) method. The Assays-on-Demand TaqMan probes and primer pair were CD177 (Assay ID Rn01454812_m1), Interleukin (IL)-17a (Assay ID Rn01757168_m1), Lipokine 2 (LCN2) (Assay ID Rn00590612_m1), IL-4 (Assay ID Rn01456866_m1), IL-13 (Assay ID Rn00587615_m1), CXCL1 (Assay ID Rn00578225_m1), IL-5 (Assay ID Rn01459975_m1), CXCL6 (Assay ID Rn00573587_g1) and IL-1β (Assay ID 00580432_m1). All experiments were performed in aStepOnePlusTM Real-Time PCR Systems (Thermo Fisher Scientific Inc., MA, USA). All expression data were normalized to endogenous control β-actin expression (Assay ID Rn00667869_m1) and calculated relative to their gene expression in each negative control.

### Histopathology

Formaldehyde-fixed left lung tissue was embedded in paraffin, sectioned at a thickness of 4 μm, and then stained with hematoxylin and eosin (HE) staining.

### Statistical analysis

Statistical analysis was carried out using IBM® SPSS® software (IBM Corporation, Chicago, IL, USA). *P* values < 0.05 were considered statistically significant. Dunnett’s test was used appropriately to detect individual differences between those rats exposed to the polypropylene samples and the controls.

### Supplementary Information

Additional file: Figure S1. Result of exposure concentrations during the inhalation exposure period. Both the lowand highconcentration groups were stable throughout the exposure period. Figure S2 Comparison of the results of neutrophil counts in BALF in nanomaterials. Inhalation exposure (**A**). Intratracheal instillation (**B**). Data are presented as mean ±SD for n= 4-5/group. These nanomaterial data are from our previous studies [14, 15]. Figure S3. Body weight following inhalation exposure (**A**) and intratracheal instillation (**B**). There were no significant changes of body weight after each exposure. Data are presented as mean ±SD for n= 4-5/group. Figure S4. Quantitative real-time polymerase chain reaction on representative inflammatory cytokine genes in lung tissue after inhalation exposure of polypropylene. There was no significant persistent increase of gene expression compared to the negative control group. Data are presented as mean ±SD for n= 5/group.

## Data Availability

The datasets during and/or analyzed during the current study are available from the corresponding author on reasonable request.
