# Supplementary figures and images for: Investigation of pulmonary inflammatory responses following intratracheal instillation of and inhalation exposure to polypropylene microplastics
Source: Part Fibre Toxicol. 2024 Aug 6;21:29. doi: 10.1186/s12989-024-00592-8 (PMC11301944; doi:10.1186/s12989-024-00592-8)

Figure S1

Figure S2


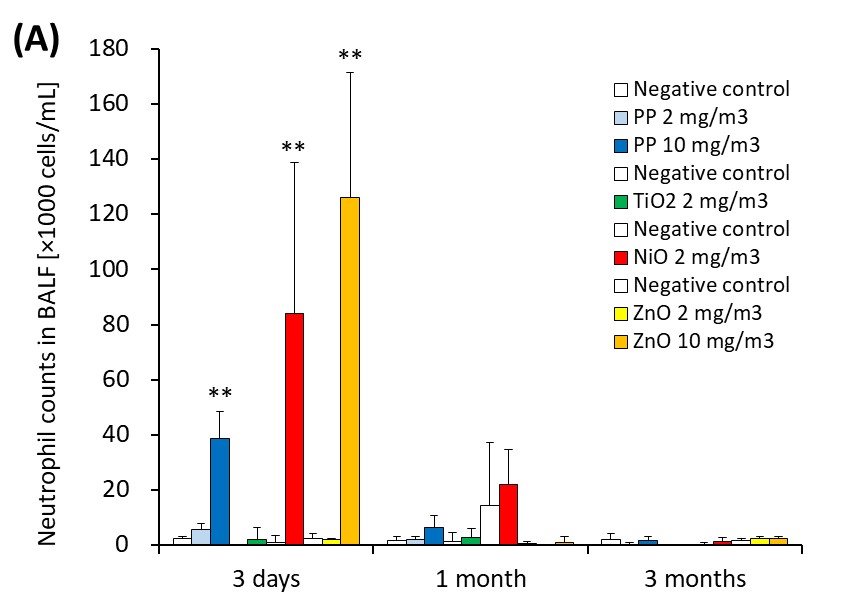


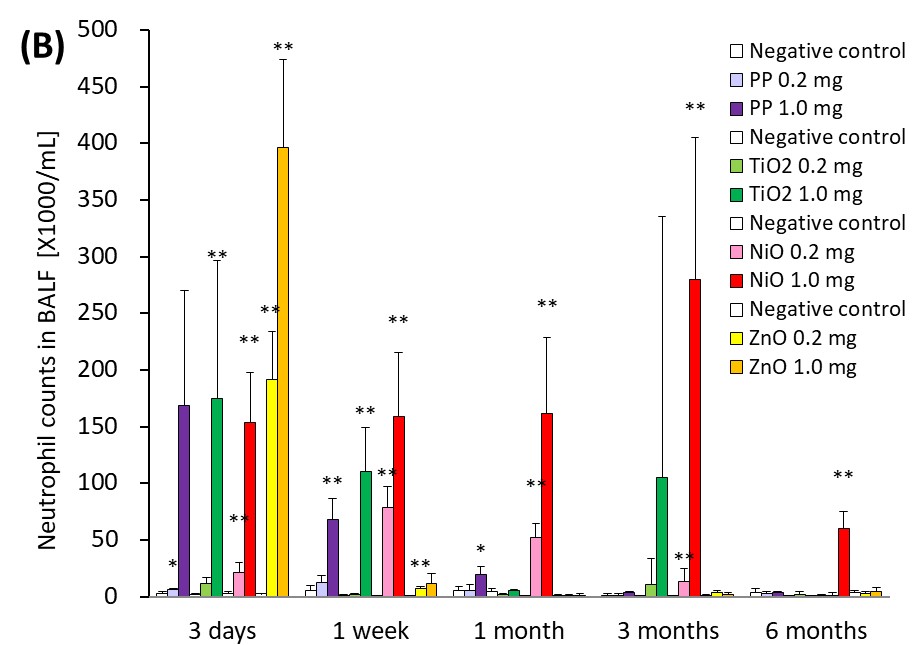


FigureS3

Figure S4

Supplement: Supplementary file 1 — Additional file: Figure S1. Result of exposure concentrations during the inhalation exposure period. Both the lowand highconcentration groups were stable throughout the exposure period. Figure S2 Comparison of the results of neutrophil counts in BALF in nanomaterials. Inhalation exposure (A). Intratracheal instillation (B). Data are presented as mean ±SD for n= 4-5/group. These nanomaterial data are from our previous studies [14, 15]. Figure S3. Body weight following inhalation exposure (A) and intratracheal instillation (B). There were no significant changes of body weight after each exposure. Data are presented as mean ±SD for n= 4-5/group. Figure S4. Quantitative real-time polymerase chain reaction on representative inflammatory cytokine genes in lung tissue after inhalation exposure of polypropylene. There was no significant persistent increase of gene expression compared to the negative control group. Data are presented as mean ±SD for n= 5/group. [file 12989_2024_592_MOESM1_ESM.docx]
